# Regulation of cGAS and STING signaling during inflammation and infection

**DOI:** 10.1016/j.jbc.2023.104866

**Published:** 2023-05-27

**Authors:** Samuel D. Chauvin, W. Alexander Stinson, Derek J. Platt, Subhajit Poddar, Jonathan J. Miner

**Affiliations:** 1 Departments of Medicine and Microbiology, University of Pennsylvania Perelman School of Medicine, Philadelphia, Pennsylvania, USA; 2 Departments of Pathology and Immunology, Washington University School of Medicine, Saint Louis, Missouri, USA; 3 Department Molecular Microbiology, Washington University School of Medicine, Saint Louis, Missouri, USA; 4 Department of Medicine, Washington University School of Medicine, Saint Louis, Missouri, USA

**Keywords:** STING, cGAS, signaling, interferon, autophagy, autoimmunity, autoinflammation, SAVI, COPA syndrome, STING-associated vasculopathy with onset in infancy

## Abstract

Stimulator of interferon genes (STING) is a sensor of cyclic dinucleotides including cyclic GMP-AMP, which is produced by cyclic GMP-AMP synthase (cGAS) in response to cytosolic DNA. The cGAS–STING signaling pathway regulates both innate and adaptive immune responses, as well as fundamental cellular functions such as autophagy, senescence, and apoptosis. Mutations leading to constitutive activation of STING cause devastating human diseases. Thus, the cGAS–STING pathway is of great interest because of its role in diverse cellular processes and because of the potential therapeutic implications of targeting cGAS and STING. Here, we review molecular and cellular mechanisms of STING signaling, and we propose a framework for understanding the immunological and other cellular functions of STING in the context of disease.

Cytosolic nucleic acid sensors are pattern recognition receptors that initiate innate immune responses against pathogens. Perhaps the most well-known cytosolic DNA-sensing pathway is the cGAS–STING pathway, which has been studied widely and has diverse cellular and immunological functions that extend beyond its role in recognizing pathogens. Indeed, cGAS-STING signaling regulates numerous cellular processes in mammalian cells including induction of the antiviral type I interferon (IFN) response, pro-inflammatory cytokine production, autophagy, senescence, metabolism, and apoptosis (1, 2, 3, 4, 5).

Stimulator of IFN genes (STING) is an endoplasmic reticulum (ER)-associated, four-pass transmembrane (TM) protein encoded by the *STING1* gene (previously *TMEM173*). Based on evidence of shared ancestry among archaea, bacteria, and eukaryotes (6, 7), STING likely evolved more than 600 million years ago (8). Ancient orthologs of STING exist in prokaryotes and invertebrates (8, 9), suggesting that STING represents a primordial mode of pathogen detection. Furthermore, numerous bacteria produce cyclic dinucleotides (CDNs), which are STING ligands (6, 10, 11, 12). Bacterial CDNs play a major role in quorum sensing and responses to bacteriophages (6, 10, 11, 12). Thus, sensors of STING and its CDN ligands represent highly evolutionarily conserved systems.

Activation of mammalian STING is triggered by an endogenous CDN second messenger (2′3′-cyclic GMP-AMP, 2′3′-cGAMP), which is produced by the enzyme cGAMP synthase (cGAS). Cytosolic DNA binds to and activates cGAS, leading to initiation of cell-intrinsic innate immune responses (13, 14). In addition to a role for STING in responding to endogenous second messengers, STING is also activated by bacterial CDNs (15, 16, 17). Upon activation, STING exits the ER and begins engaging TANK-binding kinase-1 (TBK1) and IFN regulatory factor-3 (IRF3), which are key downstream effectors (18, 19, 20) (Fig. 1).

**Figure 1:**
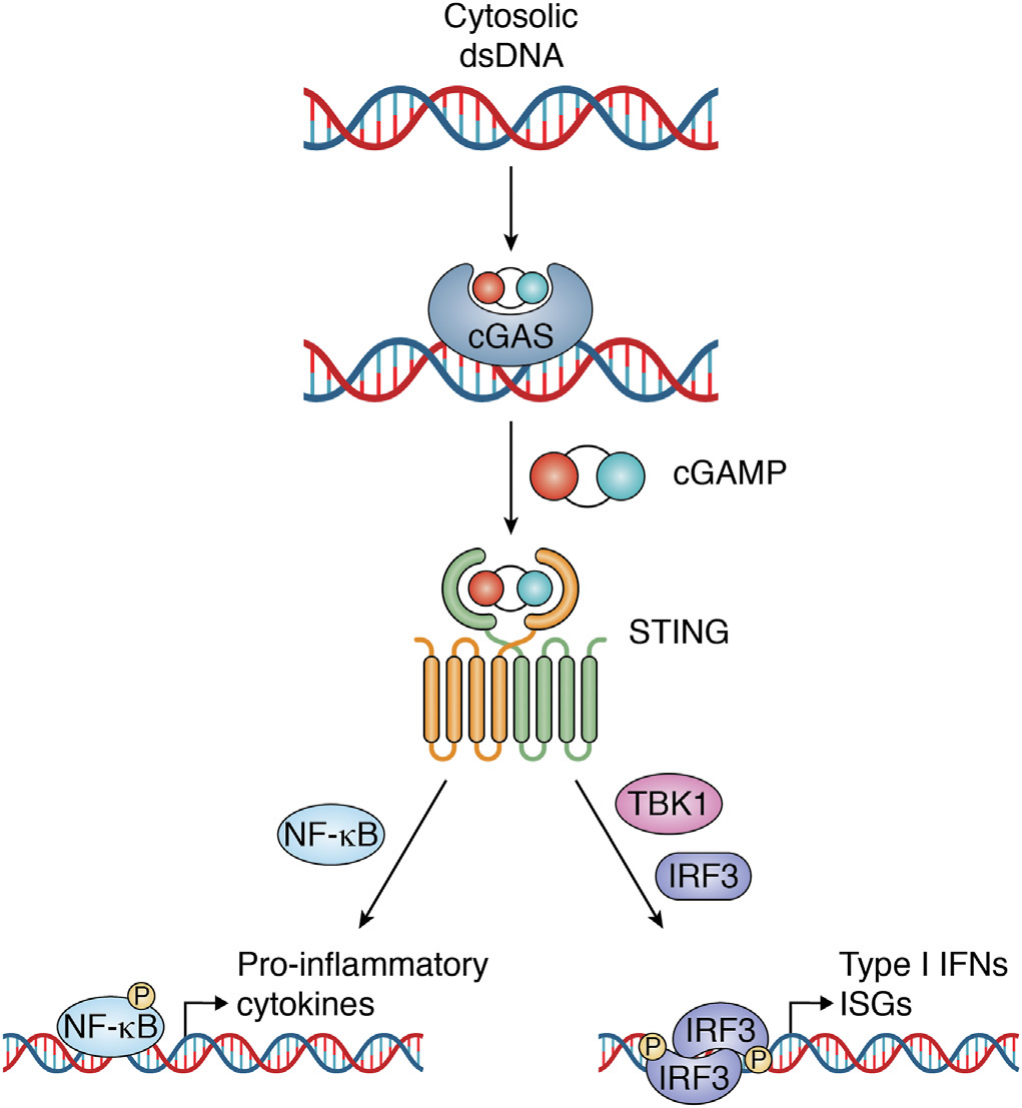
**Cytosolic DNA activates the cGAS-STING pathway.** Cytosolic dsDNA, including from viruses, bacteria, and mislocalized endogenous DNA, binds to cGAS. DNA-bound cGAS undergoes a conformational shift that triggers production of cGAMP. Dimeric STING binds to cGAMP and undergoes a conformational change, allowing recruitment of TBK and the phosphorylation of the transcription factors IRF3 and NF-κB. Phosphorylated IRF3 and NF-κB translocate to the nucleus where they facilitate the transcription of effector genes. cGAMP, cyclic GMP-AMP; IRF3, IFN regulatory factor-3; STING, Stimulator of interferon genes

The cGAS–STING pathway must be tightly regulated to prevent excessive signaling. Aberrant activation of the cGAS–STING pathway causes severe autoinflammatory or autoimmune diseases in humans and in mice. For example, STING-mediated autoimmunity occurs in humans with specific mutations in *TREX1*, *STING1*, and *COPA*, albeit with distinct molecular, immunological, and clinical phenotypes (21, 22, 23, 24). Dissecting the associated cellular, immunological, and molecular mechanisms of the cGAS–STING pathway is an active area of investigation. Here, we highlight recent advances in the field as well as some current areas of study.

## Mechanisms of cGAS activation

**Figure 2:**
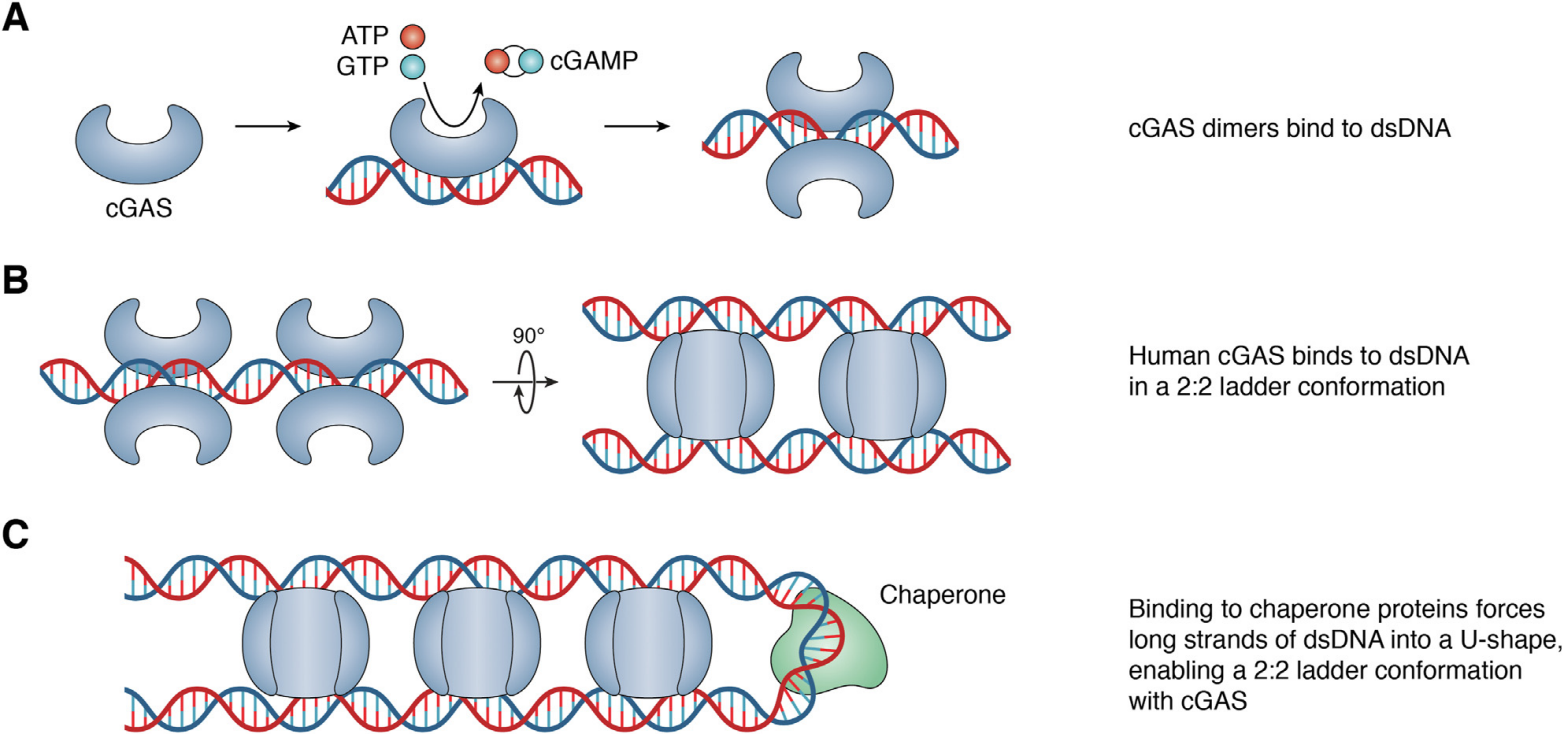
**cGAS dimers form a ladder-like structure on long dsDNA.***A*, cGAS binds to dsDNA, changing the conformation of the active site, enabling the conversion of ATP and GTP to cGAMP. Dimerization of cGAS to dsDNA stabilizes the active site. *B*, longer strands of dsDNA (>50 bp) can accommodate multiple cGAS dimers and are organized in a 2:2 ladder conformation. *C*, long strands of dsDNA can be organized into U-loops *via* chaperone proteins, allowing for cGAS dimers to stably bind. cGAMP, cyclic GMP-AMP.

The endogenous STING ligand is cGAMP, which is produced by cGAS, a bilobed horseshoe-shaped protein that is primarily localized within the cytosol (25) (Fig. 2). The active site of cGAS resides on the inside of the horseshoe between the nucleotidyl transferase–containing C-terminal and the N-terminal lobes (25). The crystal structure of the cGAS active site suggests a mechanism of cGAMP formation consistent with that of other nucleotidyl transferases, where one nucleotide attacks the other to create a dinucleotide bond (25). DNA binds to the outer edge of cGAS, on the side opposite of the active site (25) (Fig. 2*A*). DNA binding is stabilized, in part by a protruding peptide that coordinates a Zn^2+^ ion that interacts with the major groove of a dsDNA molecule (25). Following DNA binding, a conformational change in cGAS generates a loop that binds GTP and ATP in the active site, thereby facilitating 2′3′-cGAMP synthesis (26, 27, 28). The active catalytic site is stabilized by homodimerization such that each cGAS monomer binds to DNA, adopting a 2:2 cGAS:dsDNA molar ratio (27). Shorter lengths of DNA (<20 bp) only bind to one subunit of a cGAS dimer, resulting in a less stable cGAS–DNA complex and therefore less efficient activation of cGAS (27, 29, 30). Longer strands of DNA (>50 bp) promote formation of ladder-like cGAS–DNA complexes where cGAS dimers resemble rungs between dsDNA molecules (31) (Fig. 2*B*). Thus, efficient cGAS assembly and signaling can occur when cGAS dimers interact with two separate dsDNA molecules or when cGAS binds to different ends of one longer U-shaped dsDNA molecule (31) (Fig. 2*C*). Furthermore, cGAS–DNA complexes form lattice-like networks in liquid-phase condensates that amplify 2′3′-cGAMP synthesis, suggesting that multivalent cGAS–DNA interactions strengthen enzymatic activity (26, 32). cGAS can associate with the cytosolic face of endosomes where the spleen tyrosine kinase can phosphorylate cGAS to promote cGAMP production (33).

## Ligand-induced conformational changes in STING

Structure-function studies indicate that CDN ligands induce polymerization of STING, leading to recruitment and activation of downstream effectors (34). Cryo-EM of STING demonstrates that in its autoinhibited conformation, STING is primarily dimeric, consisting of a cytosolic ligand-binding domain, a tetra-spanning TM domain, and an unordered C-terminal tail (34). The 2′3′-cGAMP second messenger binds inside the V-shaped ligand-binding domain of STING (35), leading to a large conformational change (34). The ligand-binding domains rotate 180° relative to the TM domain, thereby swapping location with the other dimer (34). This results in uncrossing of the connector loops, which separate the ligand-binding and TM domains (34). The ligand-binding domain then narrows in width while simultaneously forming a tight lid-like closure over 2′3′-cGAMP within the binding pocket (34, 36).

STING can undergo both polymerization and membrane-associated clustering. The rotation of STING induced by 2′3′-cGAMP generates a polymeric interface through movement of alpha helices (34). Polymerization of STING likely initiates at the ER membrane when multiple ligand-bound STING dimers assemble side-by-side (37). Mutating residues Gln273 and Ala277 to prevent interactions at the polymeric interface is sufficient to block ER exit and subsequent STING signaling (34).

Additionally, palmitoylation of STING at cysteine 91 (Cys91) is required for STING signaling (38, 39), perhaps because of palmitoylation-dependent clustering at the *cis*-Golgi (40). Although mutation of Cys91 or inhibition of palmitoylation can block clustering, blockade of palmitoylation does not disrupt ER-to-Golgi trafficking of STING (38, 39). Thus, whereas ligand-induced polymerization of STING promotes translocation to the Golgi, palmitoylation likely mediates membrane clustering of STING, both of which are critical for signaling (34, 37).

## Regulation of STING signaling by its C-terminus

Oligomerization is a fundamental property of STING that facilitates its interactions with TBK1 and IRF3 (41). TBK1 binds to a conserved motif of the C-terminal tail of STING (42, 43). Because TBK1 is a homodimer, it interacts simultaneously with two STING molecules (42, 43). Current thinking is that a conformational change releases the STING C-terminal tail to allow recruitment and activation of TBK1 (44). Recruitment of IRF3 to the STING–TBK1 signaling complex is promoted by TBK1-mediated phosphorylation of serine 366 in the pLxIS^366^ motif of the STING C-terminal tail (18, 20). Phosphorylated IRF3 undergoes homodimerization and translocation to the nucleus, where it induces the transcription of type I IFN and other ISGs. Given the role of STING polymerization during signaling, it is possible that forced oligomerization can occur during overexpression of STING. This may explain why spontaneous STING signaling happens in the absence of 2′3′-cGAMP (1, 34).

STING activation also triggers canonical NF-κB signaling (NF-κB subunit p65; RelA). Unlike STING-mediated activation of IRF3, interruption of ER-to-Golgi STING trafficking does not abrogate NF-κB signaling (45). However, the precise mechanism by which STING induces NF-κB signaling is less well understood. One possibility is that activation of NF-κB downstream of STING may somehow involve ubiquitylation. For instance, ubiquitin scaffolding is required for NF-κB signaling in general (46), and STING is polyubiquitylated at multiple lysines (47, 48, 49, 50). However, it is less clear whether STING itself or another molecule serves as the direct scaffold on which NF-κB activation occurs downstream of STING.

Surprisingly, TBK1 is not absolutely required for STING-induced NF-κB activation. Indeed, STING can activate NF-κB signaling in the absence of the STING C-terminal tail (51, 52), which includes the TBK1-binding site. Although TBK1 is required for the full activation of both IRF3 and NF-κB in murine embryonic fibroblasts transfected with dsDNA or treated with the STING agonist DMXAA (53), it is dispensable for STING-induced NF-κB activation in bone marrow–derived macrophages (BMDMs) expressing inhibitor of NF-κB kinase subunit epsilon (IKK-ε) (19), suggesting cell type–specific STING signaling pathways. Phosphorylation of other IKK family members, including IKK-α and IKK-β, can also occur downstream of either of TBK1 or IKK-ε in murine embryonic fibroblasts, BMDMs, and HeLa cells (19, 53, 54). TAK1 (transforming growth factor β-activated kinase 1) and IKK-γ (NEMO) may act as intermediaries of the STING/TBK1/IKK-ε-IKK-α/IKK-β cascade (19, 54).

In addition to cell type–specific expression of STING signaling mediators, the type of stimulus (*e.g.*, dsDNA, exogenous CDN, or infection) may trigger distinct signaling events. For instance, DNA damage induces an atypical cGAS-independent signaling axis to induce an NF-κB–dependent transcriptional program downstream of STING (45). In that context, ATM and p53 activation mediate TRAF6-catalyzed polyubiquitylation of STING to organize the NF-κB response. In addition to canonical NF-κB activation, noncanonical NF-κB activation also can occur downstream of STING. For example, ionizing radiation induces STING and TBK1-dependent formation of the NF-κB subunit p52/RelB complex, which limits the type I IFN response (55). Thus, although there is a clear association between STING and NF-κB signaling, the scenarios and mechanisms by which STING activates NF-κB remain to be fully elucidated.

To prevent aberrant signaling, STING is autoinhibited by its C-terminal tail (52). The mechanism of autoregulation may be (1) steric hindrance of the ligand-binding pocket of STING (56) or (2) shielding of polymeric interfaces (37), but this is not entirely clear since the structure of the C-terminal tail was not visualized by crystallography (34, 56). Interestingly, genetic gain-of-function mutations in STING have been identified in humans with an autoinflammatory disease called STING-associated vasculopathy with onset in infancy (SAVI) (22). Patients with SAVI develop fever, skin lesions, digital ulcerations, and interstitial lung disease (pulmonary fibrosis) (22, 57, 58). Known SAVI-causing mutations include those found in the connector helix loop (V147L, V147M, N154S, V155M) (22, 59), in the ligand-binding pocket (C206Y) (60) and at the polymeric interface (R281Q, R284G, R284S) (58, 60, 61). In cell culture, these mutations cause spontaneous upregulation of type I IFN signaling in the absence of either cGAS or 2′3′-cGAMP (22, 62), potentially as a consequence of enhanced dimerization (22) or polymerization (37), spontaneous ER exit (62), or destabilization of the ligand-binding domain (34). These mutation-induced structural and functional phenomena may be related, and the underlying molecular mechanisms remain a topic of active study.

## Regulation of autophagy by STING

In addition to transcriptional regulation *via* IRF3 and NF-κB, STING activation can also induce autophagy. Autophagic turnover of the cytosol preserves cellular integrity by removing potentially harmful pathogens or damaged organelles. In some metazoans, including mammals and the invertebrate sea anemone *Nematostella vectensis*, STING activation induces autophagy as an antiviral defense (3). Since STING-mediated autophagy occurs independently of the STING C-terminal tail, which is notably absent in *N. vectensis*, this process likely represents a conserved, ancient biological function of STING. Indeed, STING-dependent autophagy degrades *Mycobacterium tuberculosis* (17), micronuclei or damaged DNA (63, 64), and ruptured mitochondria (65), thereby counter-regulating activation of STING by cGAS. STING also prevents energy stress-dependent autophagy (66), suggesting that the role of STING in regulating autophagy extends beyond dampening STING signaling.

**Figure 3:**
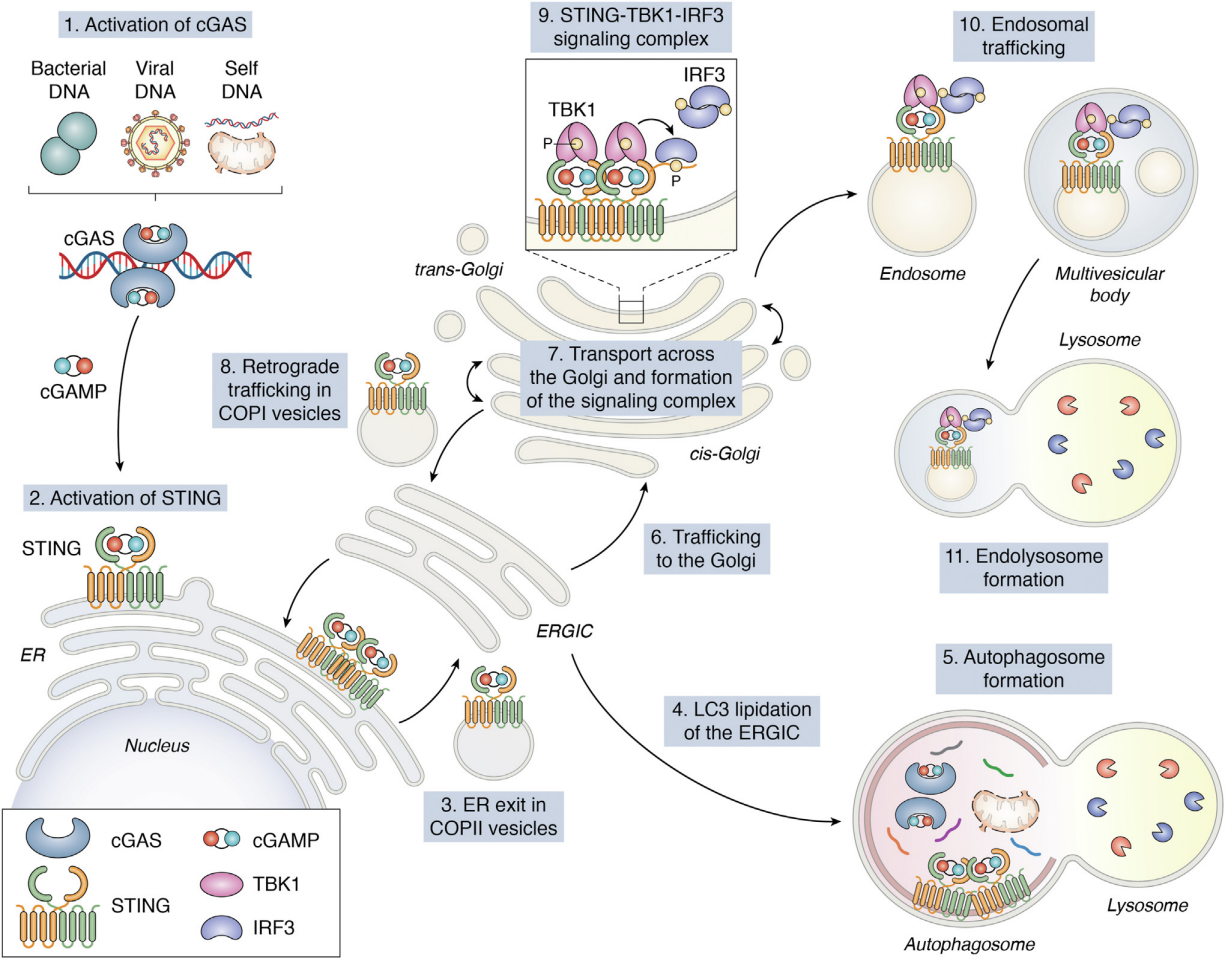
**STING is regulated by its subcellular localization.** (1) Cytosolic DNA triggers cGAS to produce 2′3′-cGAMP, which in turn (2) activates STING at the ER. (3) Polymeric STING is sorted into COPII vesicles destined for the ERGIC or Golgi. (4) At the ERGIC, STING induces LC3 lipidation of the ERGIC membrane, which leads to the generation of an autophagosome that includes STING and other molecules such as cGAS. (5) STING is negatively regulated by its own degradation in the autophagolysosome. (6) Alternatively, STING accumulates in the *cis*-Golgi. (7) STING forms a signaling complex with TBK1 and IRF3 in the *trans*-Golgi and post-Golgi vesicles. (8) STING can return from the *cis*-Golgi to the ER in COPI vesicles, or (9) the STING–TBK1–IRF3 signaling complex is fully formed at the *trans*-Golgi network. (10) The STING signaling complex is routed through endosomes and multivesicular bodies. (11) Lysosomal targeting of the STING signaling pathway results in its degradation after signaling and trafficking through the Golgi and into post-Golgi vesicles. cGAMP, cyclic GMP-AMP; ERGIC, ER–Golgi intermediate complex; IRF3, IFN regulatory factor-3; STING, Stimulator of interferon genes; TBK1, TANK-binding kinase-1.

Trafficking from the ER to the Golgi or ER–Golgi intermediate complex (ERGIC) (3, 62) is required for STING-mediated induction of autophagy (Fig. 3). During autophagy, LC3 lipidation catalyzes the elongation of the membrane, which serves as a precursor to the development of the double-membraned autophagophore. STING-induced LC3 lipidation occurs at the ERGIC and relies on the autophagy proteins WD domain, phosphoinositide interacting 2 (WIPI2) and autophagy related 5 (ATG5) (3) but requires neither the conventional unc-51–like autophagy activating kinases 1 and 2 (ULK1 and ULK2) nor class III PI3K complexes (3, 52, 67, 68). STING contains an LC3-interacting motif (residues 333–334) that facilitates LC3-I recruitment, enabling direct induction of autophagy (3, 68). Hence, STING-induced autophagy is uncoupled from STING-induced activation of the type I IFN response (3, 67, 68). However, mouse macrophages expressing STING without its C-terminal tail (ΔCTT STING, STING V340X) cannot activate LC3 lipidation or aggregation in response to cGAMP stimulation (69), suggesting a role of the C-terminal tail in autophagy regulation. Further studies of ΔCTT STING mice may help to delineate the physiological relevance of STING-induced autophagy.

One effect of STING-dependent autophagy is its own counter-regulation. Both cGAS and STING are degraded in autophagosomes (3, 67, 68, 70, 71). STING is directly encapsulated into autophagic vesicles by the endosomal sorting complexes required for transport proteins (71, 72). Additionally, the ubiquitin adapter p62 recognize ubiquitin scaffolds on cGAS and STING (67, 68, 70, 71, 73), recruiting them to the autophagosome. p62 is activated by TBK1 (65, 67, 74), suggesting that STING interacts with p62 at the Golgi. However, STING requires vesicular trafficking from the ERGIC to enter the autophagosome (3). Retrograde trafficking of STING to the ERGIC for autophagic degradation (67) may occur independently of lysosomal trafficking of STING through post-Golgi compartments, but the precise mechanisms remain to be determined.

## Regulation of STING by trafficking and lysosomal degradation

Whereas retrograde trafficking of STING plays a role in autophagic degradation, anterograde movement of STING from the ER to the Golgi in COPII vesicles is essential for induction of the type I IFN response (Fig. 3). In general, COPII vesicle formation begins through SAR1A-mediated assembly of the SEC23-SEC24 heterodimer and SEC13-SEC31 tetramer that together form a coatomer cage (75, 76). Knockdown of SAR1A or the SEC23-24 heterodimer prevents signaling downstream of STING (3, 77). Additionally, STING co-immunoprecipitates with SEC24C (3, 78, 79), which is the primary subunit responsible for binding to membrane cargo proteins and concentrating them into COPII vesicles (76). Other proteins likely contribute to ER exit of STING, including YIP family 5 (YIPF5) and transmembrane emp24 protein transport domain containing 2 (TMED2), although it is unclear whether these molecules directly promote interactions between SEC24C and STING (77, 79). Furthermore, entry of STING into COPII vesicles is a likely dynamic process regulated by multiple ADP-ribosylation factor (ARF) GTPases (3, 62).

Posttranslational modifications of STING at the ER also regulate its trafficking. For example, polyubiquitylation of STING occurs at the ER, and elimination of specific polyubiquitylated lysine residues increases ER localization of STING, suggesting diminished ER export (50, 80). STING mutations that disrupt the polymeric interface also prevent ER-to-Golgi trafficking (37). Thus, STING is likely polymeric when loaded into the COPII vesicle (34, 37), and ubiquitylation might regulate sorting of STING into COPII vesicles.

The activated STING signaling complex has been detected within multiple cellular compartments. For instance, STING can complex with IRF3 at the ERGIC after disruption of ER-to-Golgi trafficking (62, 81). Since immunofluorescence experiments indicate that the STING–TBK1 signaling complex forms at the Golgi (38, 82), the presence of activated signaling complexes within the ERGIC might reflect retrograde transit of the STING signaling complex. Although it remains possible that the signaling complex forms at the *cis*-Golgi and is maintained as it moves through the Golgi stacks, other studies strongly suggest that STING signaling is initiated later, at the *trans*-Golgi network (38, 44, 83).

Currently, two models are proposed for intra-Golgi trafficking in general. In one model, *cis*-Golgi cisternae undergo maturation to become *trans*-Golgi cisternae, which are gradually degraded by incremental endosomal assembly (84). A second model is that COPI vesicles bidirectionally bleb from cisterna to cisterna (85). COPI vesicles mediate retrograde trafficking to the ER through ARF1-mediated recruitment of the COPI heptameric coat (α-, β-, β’-, γ-, δ-, ε-, ζ-COP) *en bloc* at the Golgi (86, 87).

Disruption of COPI-mediated trafficking leads to constitutive STING signaling. For instance, mutations in *COPA* (α-COP) cause COPA syndrome, a rare autoimmune disease characterized by enhanced STING signaling (24, 82, 88, 89, 90). Although mutant COPA likely impacts trafficking of many molecules, genetic deletion of STING is sufficient to ameliorate disease in mice that express a human disease-causing COPA mutation (88, 89). COPA mutants exhibit diminished interaction with SURF4, a COPI cargo receptor, which may explain impaired retrograde trafficking of STING in COPA mutant cells (82, 88, 89). Indeed, deletion of either SURF4 or COPA triggers spontaneous STING signaling (82, 89). Furthermore, deletion of SURF4 spontaneously upregulates STING signaling in *cGAS*^*−/−*^ cell lines (82). Thus, STING undergoes homeostatic ER-to-Golgi trafficking in the absence of endogenous ligand, and retrograde trafficking of STING is necessary to prevent spontaneous STING signaling.

Activated STING is degraded in the lysosome, leading to termination of cGAS-STING signaling (72, 91, 92). Lysosomal targeting of STING depends on the lysosomal membrane protein Niemann-Pick type C1 (NPC1) (91). Indeed, NPC1 mutant cells from patients with Niemann-Pick disease type C exhibit enhanced STING signaling, in part, because degradation of STING is partially inhibited (91). Thus, STING localization is an essential feature of appropriate STING signaling, and mislocalization of STING leads to spontaneous inflammation.

## Regulation of antimicrobial immunity by cGAS and STING

A seminal paper in 2008 reported that STING mediates immunity to diverse pathogens including a DNA virus (herpes simplex virus 1; HSV-1), an RNA virus (vesicular stomatitis virus), and an intracellular bacterium (*Listeria monocytogenes*) (1, 93). Bacterial CDNs were already known to cause inflammation although the receptor had not yet been identified (94, 95, 96). CDNs were later found to be the stimulatory ligands of STING (97). Indeed, bacterial CDNs directly bind to STING and induce the type I IFN response (97, 98). Since viruses do not produce CDNs, it was not until the discovery of cGAS that the role of cGAMP was elucidated as the endogenous ligand for STING (13, 14, 99, 100).

IFN production and signaling are well known to limit viral replication and spread. Indeed, IFN production induced by acute STING activation protects cells against severe acute respiratory syndrome coronavirus 2 (SARS-CoV-2) infection and protects mice against both SARS-CoV-2 and HSV-1 infection (101, 102). Furthermore, IFN production from the cGAS–STING pathway protects against retroviruses including human immunodeficiency virus, murine leukemia virus, and simian immunodeficiency virus (103). Retroviruses are RNA viruses that produce DNA products during their replication cycle (104). However, other RNA viruses do not produce DNA intermediates, yet genetic deletion of STING still makes mice more susceptible to infection (93, 105, 106). Although the exact mechanisms are likely complex, one compelling hypothesis is that RNA viruses activate cGAS *via* release of endogenous mitochondrial DNA (mtDNA) in infected cells (1, 107, 108, 109, 110). Indeed, the cGAS–STING pathway is activated by cytosolic mtDNA (110, 111, 112).

**Figure 4:**
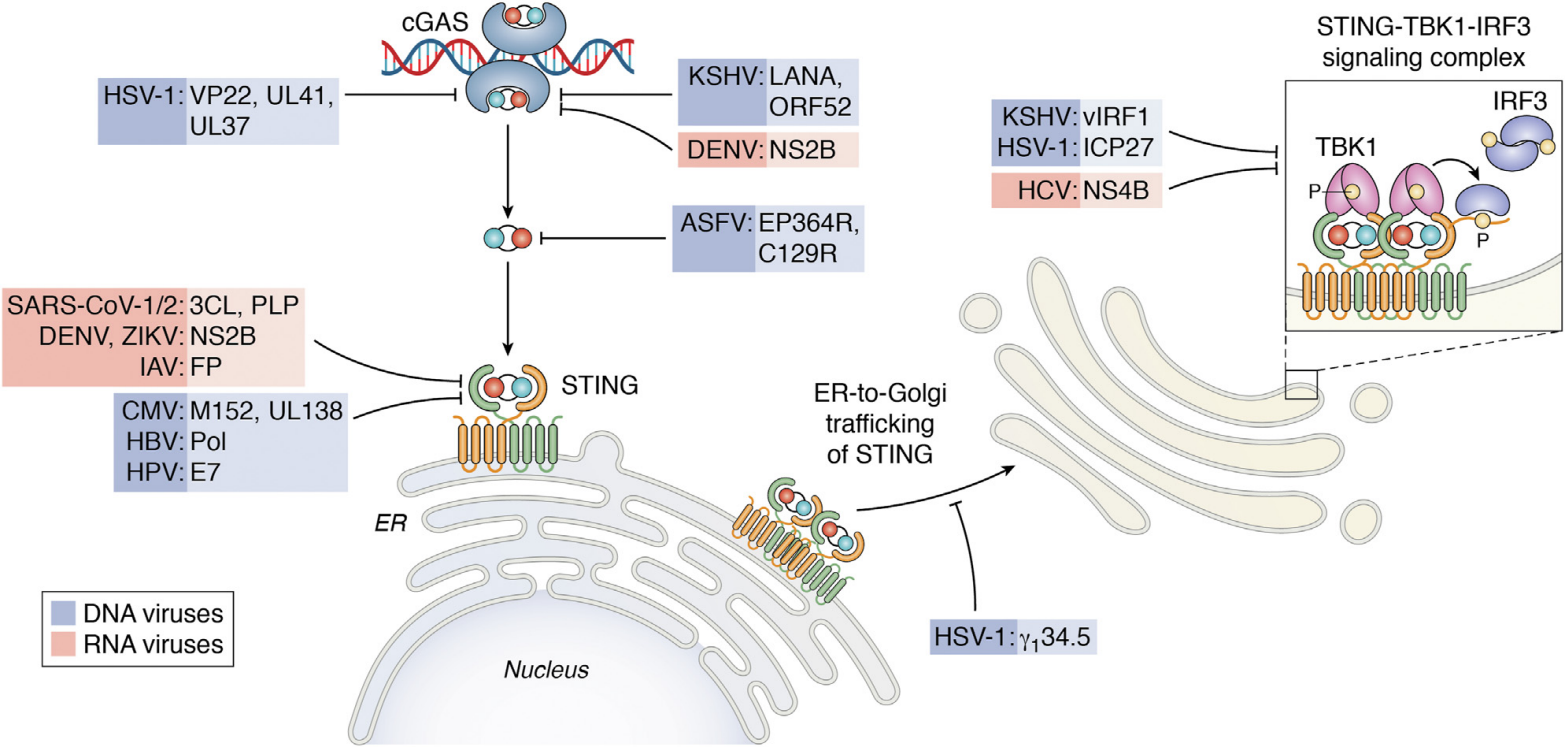
**Diverse mechanisms of viral subversion of cGAS-STING signaling.** Both DNA and RNA viruses have developed various and sometimes similar mechanisms to antagonize cGAS-STING signaling. Depicted is the cGAS-STING pathway with RNA and DNA viruses, as well as corresponding viral proteins which inhibit signaling at each stage of the pathway. STING, Stimulator of interferon genes.

Highlighting its essential role in preventing cellular infection and replication, many DNA and RNA viruses have co-evolved mechanism to antagonize the cGAS–STING pathway (Fig. 4). Herpesviruses HSV-1 and Kaposi sarcoma herpesvirus both produce proteins that bind to and inhibit cGAS (113, 114, 115, 116). Additionally, the nonstructural protein 2B (NS2B) protease of the flavivirus Dengue virus degrades cGAS (108). The arbovirus African swine fever virus produces two phosphodiesterases that cleave cGAMP (117). STING is also directly inhibited by viral proteins encoded by the DNA viruses cytomegalovirus and human papilloma virus (118, 119, 120). Flaviviruses including West Nile virus (WNV), Zika virus, and Japanese encephalitis virus induce NS2B-mediated STING degradation (108, 121, 122). STING ubiquitination is also targeted by viral polymerase (Pol) of hepatitis B virus and the papain-like proteases of SARS-CoV-1 and SARS-CoV-2 (123, 124, 125, 126). Influenza A virus, on the other hand, blocks STING dimerization (127). HSV-1 also disrupts another step in STING signaling: trafficking from the ER to Golgi (128). Lastly, proteins encoded by Kaposi sarcoma herpesvirus, HSV-1, and hepatitis C virus disrupt the interactions between STING, TBK1, and IRF3 in the STING signaling complex (129, 130, 131, 132). Certainly, the cGAS–STING pathway played an influential role in the evolution of many types of viruses.

The cGAS–STING pathway also plays important immunomodulatory roles beyond IFN and virus-infected cells. Despite the ability of HSV-1 to inhibit cGAS activation, block STING trafficking, and inhibit the STING signaling complex, HSV-1 infection still leads to increased death in mice with genetic deletion of either cGAS or STING (93, 133). Similarly, STING deletion causes impaired survival following infection with WNV (106). Intriguingly, SAVI mice with chronic STING activation also have decreased survival following infection with WNV or murine gammaherpesvirus 68 (γHV-68) (106). SAVI mice have major defects in both innate and adaptive immunity (23, 106, 134), highlighting a role of STING in immune regulation in addition to antiviral immunity. Indeed, STING agonism in activated T cells induces T cell apoptosis and anergy and inhibits formation of T cell memory (135, 136). Thus, although STING activation may be necessary to mount an appropriate antiviral response, excessive STING activation also impairs immune function.

The cGAS–STING pathway also impacts antibacterial immunity. Although cGAS can be activated by bacterial DNA (16) and STING can be activated by bacterial-derived CDNs (16, 137), the roles of cGAS and STING during bacterial infection are complicated. For example, while cGAS-deficient mice are more susceptible to *M. tuberculosis* (*Mtb*) infection, STING-deficient mice are not (138). Thus, cGAS exerts some STING-independent effects during *Mtb* infection. Furthermore, whether STING is protective or deleterious during bacterial infection depends on the pathogen. Although genetic deletion of STING leads to impaired control of the intracellular bacteria *L. monocytogenes*, *Chlamydia trachomatis*, and *Salmonella enterica* (93, 137, 139), in the cecal ligation and puncture model of sepsis, loss of STING is protective against death and bacteremia (140, 141, 142). Additionally, STING KO mice have reduced immunopathology in *Helicobacter pylori* infection (143). Thus, the effect of STING on antibacterial immunity is pathogen- and cell type–specific.

One of the challenges to understanding STING activation by bacterial CDNs is the plasma membrane barrier, which exogenous CDNs must cross to become accessible to cytosolic STING. Although the bacterial CDNs c-di-GMP and c-di-AMP can potently stimulate mouse monocytes and macrophages (94, 144), these molecules are bulky and charged, making them unlikely to enter the cytosol by simple diffusion. Both pharmacologic inhibition of clathrin-mediated endocytosis and genetic loss of the membrane pore protein folate transporter 1 (SLC19A1) dampen CDN-induced IFN responses in myeloid cells (144, 145, 146). Thus, bacterial CDNs can access the cytosol by clathrin-mediated endocytosis and by crossing a plasma membrane pore. Additionally, the ATP-binding cassette transporter ABCC1 can export cGAMP out of a cell, suggesting a mechanism for STING stimulation in neighboring cells after cGAS activation (147).

STING is not the only CDN-binding protein that regulates host immune responses. For example, the bacterial cyclic dinucleotide c-di-AMP inhibits the murine protein reductase controlling NF-κB (RECON), which is itself an inhibitor of NF-κB–mediated inflammation (148). Since RECON binds to bacterial CDNs with greater affinity than STING, RECON functions as a molecular sink that blunts STING-mediated IRF3 activation in favor of increased NF-κB signaling (148). Indeed, inhibition of RECON by c-di-AMP leads to increased NF-κB activity and restricts *L. monocytogenes* in murine macrophages (148). Therefore, careful balancing of STING signaling with other antimicrobial signaling is necessary for appropriate responses at both the cellular and animal levels during infection.

## Role of endogenous DNA in cGAS-STING activation

Interestingly, cGAS-STING–mediated protection against many intracellular pathogens including RNA viruses and *S. enterica* is hypothesized to be driven by the detection of mtDNA released into the cytoplasm upon infection (1, 107, 108, 139), which emphasizes the potential for cGAS-STING activation by endogenous DNA. Typically, the nuclear envelope and mitochondrial membrane act as barriers to prevent cytosolic detection of endogenous DNA. However, pathology can result when these barriers break down. During interphase, nucleases are positioned throughout the cell to degrade cytosolic DNA (149, 150). Thus, three prime repair exonuclease 1 (TREX1) and DNaseII prevent activation of the cGAS–STING pathway from DNA that escapes the nucleus or mitochondria (151, 152). During mitosis, the nuclear membrane breaks down (153), leading to interactions between cytosolic proteins and nuclear DNA. Throughout this process, posttranslational modifications downregulate cGAS–STING signaling. For example, cGAS is hyperphosphorylated by Aurora kinase B to reduce its capacity to produce cGAMP (154), and cGAS is also polyubiquitylated to trigger its degradation in the autophagolysosome (70, 80). Thus, numerous mechanisms regulate cGAS and cGAMP-mediated STING signaling in the context of mitosis and in other scenarios where the nuclear and mitochondrial membranes are disrupted.

When these control mechanisms break down, the loss of tolerance to endogenous DNA leads to unregulated cGAS-STING signaling resulting in a rare autoinflammatory type I interferonopathy called Aicardi-Goutières syndrome (AGS). Genetic loss of TREX1 and SAM domain and HD domain-containing protein 1 (SAMHD1) both lead to AGS, although the precise mechanism is poorly defined. One model of AGS is the accumulation of endogenous retroelements in TREX1- or SAMHD1-deficient cells, which activate cGAS-STING (155, 156, 157, 158, 159, 160, 161, 162, 163). Another model for AGS pathogenesis highlights the accumulation of cytosolic ssDNA from chronic DNA damage (150). Although cGAS is poorly activated by ssDNA (28), ssDNA fragments may self-anneal, forming dsDNA cGAS ligands (164). Genetic deficiency of SAMHD1 produces cytosolic ssDNA in the context of a chronic DNA damage response (157, 165, 166, 167). Similarly, TREX1-deficient cells exhibit accumulation of cytosolic ssDNA accompanied by chronic activation of DNA damage checkpoint signaling (149). Resection of damaged DNA can liberate ssDNA fragments during the repair process (168). *Trex1*^*−/−*^ cells were observed to deplete their nuclear reserves of RPA and RAD51, proteins that bind ssDNA and activate the ATR ssDNA repair pathway (150). This suggests that TREX1 deficiency may contribute to genomic instability *via* effects on DNA damage repair pathways (150). Indeed, CRISPR screens have identified a role for TREX1 in double strand break repair (169). Furthermore, DNA damage results in the formation of micronuclei that activate cGAS (170, 171, 172, 173, 174), and reactive oxygen species increase STING-inducing apoptosis (175). Hence, dysregulation of endogenous DNA can lead to aberrant cGAS-STING activation and produce a type I IFN response in the absence of infection.

## Role of chronic STING activation in disease

Both humans and mice with gain-of-function STING mutations have an autoinflammatory disease called SAVI (22, 176, 177, 178), and mouse models provide the opportunity to test underlying immunological mechanisms of disease in SAVI. STING gain-of-function mice (SAVI mice) have an autoinflammatory disease with failure to thrive, T and NK cell cytopenia, lung inflammation, splenomegaly, absent lymph nodes, neuroinflammation, and premature death (19, 23, 106, 134, 179, 180, 181, 182, 183, 184). Despite a large reduction in total numbers of T cells in SAVI mice, αβ T cells still play a major role in lung disease in STING N153S animals (134).

Observations that SAVI patients exhibit elevated ISGs and type I IFN have led to the characterization of SAVI as a type I interferonopathy (185, 186, 187). However, autoimmune lung disease in STING N153S mice develops independently of cGAS (134), IFNAR1 (134, 181, 182), IRF3 (23, 134, 181), and IRF7 (134). Surprisingly, deletion of the type II IFN receptor (IFNGR1) but not the type I or type III receptors (IFNAR or IFNLR1, respectively) diminished the severity of lung disease and restored lymph node formation in SAVI mice (188, 189). Similarly, BMDMs from SAVI mice are hyperresponsive to IFN-γ, but not to IFN-α or IFN-λ (188). Thus, chronic STING activation leads not only to increased inflammation but also to increased sensitivity to inflammatory signals. Although IFNGR1 signaling seems to drive some aspects of SAVI disease, increased IFN-γ has not been seen in these animals (134, 188), raising the possibility that SAVI cells are hyperresponsive to homeostatic cytokines.

## Conclusion

Despite a wealth of literature on the role of cGAS-STING signaling in autoimmunity and antimicrobial immunity, there are numerous open questions about the biochemistry, cell biology, and regulatory mechanisms associated with STING signaling and STING-mediated immunity. Understanding the cell type–specific effects of STING activation has become increasingly important, especially because STING agonists are undergoing clinical testing as adjuvants to prime the immune system during checkpoint blockade for cancer therapy (190, 191, 192). Although genetic gain-of-function mutations in STING cause inflammation and immunodeficiency in model systems, it is less well understood whether repeated treatment with STING agonists will provide therapeutic benefit in cancer and other human diseases or if long-term STING agonism might create immunological defects such as those observed in SAVI. Given the complex relationships between cGAS and STING structure and function, as well as the cell type–specific effects of this pathway, pharmacological targeting of these receptors will likely become increasingly important in medicine. This underscores the importance of ongoing work to decipher molecular and cellular mechanisms of cGAS-STING activation and signaling.

## Conflict of interest

The authors declare that they have no conflicts of interest with the contents of this article.
